# Discharge Mode Enhances the Energy Efficiency of Radiofrequency Microneedle: An In Vivo Porcine Study of Optimized Histological Responses Under Constant Total Energy

**DOI:** 10.1111/jocd.71158

**Published:** 2026-08-23

**Authors:** Jiayi Feng, Xuelai Wang, Ling Zhang, Shize Ma, Lvping Huang

**Affiliations:** ^1^ Asian Academy of Anti‐Aging Research and Translational Medicine (Shenzhen) Co. Ltd Shenzhen China; ^2^ Department of Plastic Surgery Shenzhen Hospital of Southern Medical University Shenzhen China; ^3^ Laser Aesthetic Center, Plastic Surgery Hospital, Peking Union Medical College and Chinese Academy of Medical Sciences Beijing China; ^4^ Research Center, Plastic Surgery Hospital, Peking Union Medical College and Chinese Academy of Medical Sciences Beijing China

**Keywords:** histological reaction, insulated, non‐insulated, radiofrequency microneedle, thermal injury

## Abstract

**Objective:**

To explore the immediate histological effects of insulated (IMRF) and non‐insulated (NMRF) radiofrequency microneedles across different discharge modes under a constant total energy, and to evaluate long‐term collagen remodeling specifically in the classical discharge mode.

**Methods:**

The backs of Bama pigs (*n* = 3) were assigned to the classical and sequential discharge modes under IMRF and NMRF treatments at a fixed total energy (1000 mJ). The classical discharge mode group was set as follows: high power and short pulse width (Group A), medium power and medium pulse width (Group B), low power and long pulse width (Group C), and the sequential discharge mode group (ultra‐high power and ultra‐short pulse width). IMRF and NMRF were used for treatment. The microthermal zone (MTZ) was evaluated and quantified by hematoxylin and eosin (HE) staining immediately after the treatment. Three months after the procedure, the skin lesions were extracted, and the mRNA expression of collagen type I and III was quantitatively evaluated by real‐time PCR.

**Results:**

Under the classical discharge mode with constant total energy delivery, a higher power level in IMRF resulted in greater MTZ density (*p* = 0.014), whereas no significant differences were observed in the depth and width of sub‐damage lesions and coagulation zones. In contrast, NMRF failed to produce typical MTZs under any of the three parameter settings. At the 3‐month follow‐up, type I collagen expression was significantly upregulated in both A and B groups of IMRF (I‐A *p* = 0.002, I‐B *p* = 0.001), while type III collagen expression showed no notable change among all groups (*p* = 0.671). Under the sequential discharge mode, a sharp increase in single‐needle peak power led to the formation of dense MTZs in both IMRF and NMRF. Compared with the medium‐power classic mode in the classical discharge mode, the sequential discharge mode resulted in significantly higher MTZ density (IMRF *p* = 0.002; NMRF *p* = 0.011). Furthermore, the sub‐damage lesion depth was significantly greater with IMRF than with NMRF (NMRF vs. I‐B *p* = 0.017; NMRF vs. IMRF *p* = 0.01).

**Conclusion:**

MTZ formation is highly dependent on the per‐needle power threshold rather than the total energy applied, and is closely associated with the upregulation of type I collagen. The sequential discharge mode, via a “high‐power ultrashort pulse width” mechanism, effectively overcomes the activation thresholds of both IMRF and NMRF without increasing the overall thermal load. By generating denser and more stable MTZs, it exhibits promising clinical potential, representing a pivotal direction for future MRF optimization.

## 1 Introduction

Radiofrequency microneedle (MRF) utilizes microneedle arrays to generate the microthermal zones (MTZ) within the dermis [1, 2], thereby promoting collagen remodeling and has been widely adopted in skin rejuvenation therapies [3, 4]. Current clinical practice primarily involves selecting either insulated microneedles (IMRF) for precise targeted heating or non‐insulated microneedles (NMRF) for broad volumetric heating based on therapeutic requirements [5, 6]. In pursuit of more pronounced skin rejuvenation outcomes, clinicians often incline to elevate the total energy output of MRF. Nonetheless, the accumulation of total thermal energy inevitably heightens the risk of adverse effects, such as prolonged post‐procedural erythema, severe pain, and post‐inflammatory hyperpigmentation (PIH). Consequently, the primary clinical challenge confronting MRF lies in how to induce sufficiently deep and dense MTZ to stimulate collagen remodeling without increasing the overall thermal burden on tissues.

Previous literature has focused on the histological changes in individual power and pulse width, without controlling for a constant total energy [6, 7], the true tissue damage threshold was masked. Based on this, this study has for the first time developed and validated the histological advantages of the sequential discharge mode. This innovative MRF electrode allocation mechanism, with ultra‐high power and ultra‐short pulse width, could break down the instantaneous tissue impedance, thereby achieving better dermal remodeling effects at a lower energy cost.

In this study, porcine models were selected as experimental subjects due to their skin tissue structure being the closest to that of humans. Initially, histological changes resulting from different combinations of power and pulse width were investigated under a fixed total energy in the classical discharge mode. Subsequently, we further examined the differences in histological outcomes between the conventional discharge mode and an innovative sequential discharge mode under equivalent parameter settings. The characteristics of thermally induced lesions generated by MRF within the skin, including morphological features and quantitative data, were systematically analyzed. These findings contribute to a deeper understanding of the histological effects of MRF, provide a robust theoretical foundation for clinical applications, and supply reliable histological evidence for the future development of MRF devices.

## 2 Methods

### 2.1 Experimental Animals

Three male Bama miniature pigs (10–11 months old; 16–17 kg) were used in this study. The Ethics Committee of Plastic Surgery Hospital, Peking Union Medical College and Chinese Academy of Medical Sciences approved the study [2023(80)]. All experiments were conducted in accordance with the approved guidelines.

### 2.2 Experimental Equipment

Radiofrequency microneedling equipment (Peninsula Medical Group, Shenzhen, China) was used in this experiment, equipped with a handpiece for the classical discharge mode and a handpiece for the sequential discharge mode. Both treatment heads were a 7 × 7 rectangular matrix consisting of 49 MRFs.

The two discharge modes of the MRF are as follows (Video 1). The classical discharge mode is most commonly used in most MRFs. In the classical discharge mode, the electrode combination consists of RF+ and RF− to form a high‐frequency current loop, and the high‐frequency energy is distributed to the 49 electrode pins simultaneously. Therefore, the power per pin is 1/49 of the set power value, while the pulse width per pin is equal to the set total pulse width. However, in sequential discharge mode, the electrodes are assigned to only one electrode pin RF+ and one electrode pin RF− for each discharge, totaling 25 groups, and each group delivers energy a total of 25 times in sequence. Therefore, the power of each pin is 1/2 of the set power value and the pulse duration of each pin is 1/25 of the set total pulse width. The energy level of each pin is calculated as: (power × pulse width)/the number of pins.

**VIDEO 1:** Video of different discharge modes. Video content can be viewed at https://onlinelibrary.wiley.com/doi/10.1111/jocd.71158.

### 2.3 Study Design

**FIGURE 1:**
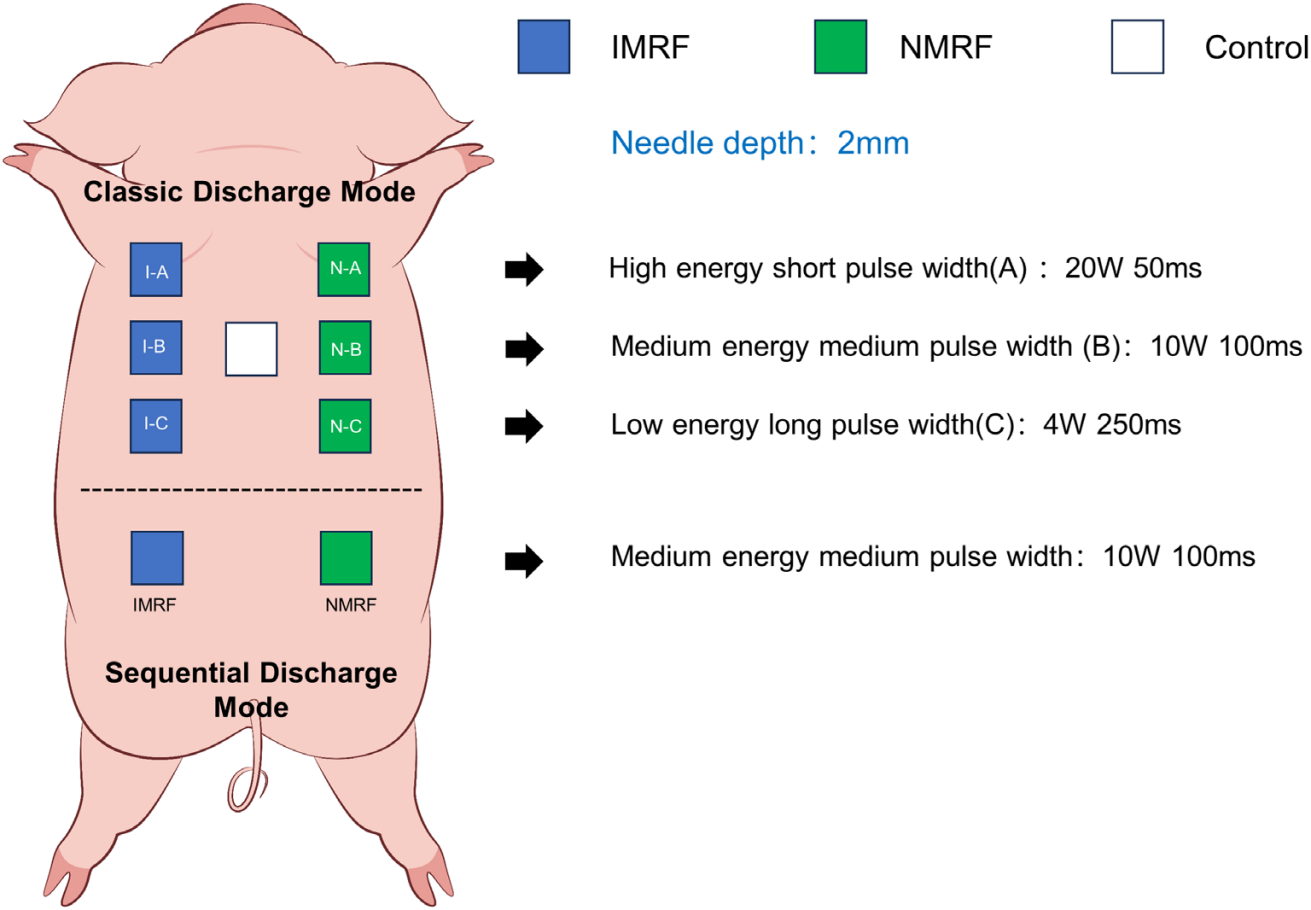
Schematic diagram of the experimental design for pigs.

Two treatment areas were designed on the back of the pig, as shown in Figure 1. The classic discharge mode area was designed on the upper side and the sequential discharge mode area was designed on the lower side, both divided into IMRF and NMRF areas. The following basic parameters remain the same in each group: the depth of the microneedle is set to 2.0 mm, three treatment passes, and the total energy per shot is controlled to 1000 mJ. In the classical discharge mode, a high power with short pulse width (A), a medium power with medium pulse width (B), and a low power with long pulse width (C) were set for IMRF and NMRF, respectively. In the blank group, the microneedle was only injected without releasing any energy. The histological and molecular changes were examined immediately and 3 months after treatment. In the sequential discharge treatment, only the medium power with medium pulse width (10 W, 100 ms) was used for both IMRF and NMRF for comparison with the classical discharge mode (Group B, 10 W, 100 ms). The specific treatment parameters of the individual regions are listed in Table 1.

**TABLE 1:** Parameters of MRF treatment.

Group	Power (W)	Pulse width (ms)	Power per pin (W/pin)	Pulse width per pin (ms/pin)	Energy level per pin (mJ/pin)
*Classic discharge mode*					
A	20	50	0.4	50	20
B	10	100	0.2	100	20
C	5	200	0.1	200	20
*Sequential discharge mode*					
IMRF	10	100	5	4	20
NMRF	10	100	5	4	20

The animals were fasted 12 h before surgery and general anesthesia was administered before the procedure. Anesthesia was administered by intramuscular injection of 0.1 mg/kg tiletamine and zolazepam (Zoletil 50, VIRBAC, FR), intubated, connected to an anesthesia machine, and maintained with 1%–1.5% isoflurane. The physiologic monitor was connected to monitor blood oxygen and heart rate, and the ear vein was implanted with an indwelling cannula. The surgical site was shaved, skinned, and cleaned. Each treatment area was 3 × 3 cm in size, tattooed with black ink, and evenly distributed on the back of each pig to ensure that each treatment area was far enough away to reduce the effects of other treatment groups. The pigs' skin reaction was assessed and photographed before and after the treatment. The entire skin layer and a portion of the subcutaneous tissue were removed from the sample area of each treatment group, and the wound was sutured with a 2‐0 nylon thread. 0.5 g ceftriaxone sodium (North China Pharmaceutical Co. Ltd., Hebei Huamin Pharmaceutical Co. Ltd.) for intramuscular injection was used for 1 week after the procedures, and 4 mg/kg tofenac (Veron, FR) was also used for intramuscular injection for postoperative analgesia for 3 days. The pigs were euthanized at the end of the entire experiment.

### 2.4 Macroscopic Examination and Histological Analysis

Samples were fixed in 10% buffered formalin, then embedded in paraffin and sectioned at a thickness of 4 μm. Because MTZs are three‐dimensional conical structures, random single cross‐sections may not accurately reflect their maximum dimensions. Therefore, 20–30 consecutive serial sections were taken from each sample area. The section displaying the deepest and widest lesion profile was methodically identified to represent the true central axis of the MTZ, ensuring accurate quantitative measurements of the maximum depth and width for statistical analysis. Hematoxylin and eosin (HE) staining was used for histological observation of the skin. Motic EasyScan Pro 6 was used to scan the sections panoramically, including measurement of the damaged lesions, sub‐damaging lesions, and the coagulation zone.

### 2.5 Real‐Time Polymerase Chain Reaction

The skin samples of the individual parameter treatment groups in the classic discharge mode were taken before, 1 and 3 months after treatment. The Sevenfast Total RNA Extraction Kit (Sevenbio, Beijing, China) was used to isolate total RNA from milled skin tissue. The HiScript III RT SuperMix (Vazyme Biotech Co. Ltd., Jiangsu, China) was used to synthesize cDNA from total RNA. Then SYBR‐Green PCR kit (Servicebio technology. Ltd, Wuhan, China) was incubated with cDNA and gene‐specific primers for real‐time PCR analysis. The 2^−ΔΔCt^ method was used for relative quantification, with GAPDH serving as the internal reference gene. The primers used in this study are listed in Table 2.

**TABLE 2:** PCR primers.

Primer	Primer sequence (5′–3′)
COL1A2	GTGCCTAGCAACATGCCAATC AGCAAAGTTCCCGCCAAGA
COL3A1	CCTCATTAGTCCCGATGGTTCT AACAGTAGAAGGACTGGCACTTATG
GAPDH	GACATCAAGAAGGTGGTGAAGCA GTCGTACCAGGAAATGAGCTTGA

### 2.6 Statistical Analysis

SPSS 26.0 software was used to analyze the data. The individual animal (*n* = 3 pigs) served as the experimental unit for all statistical analyses. The results are given as mean ± SD. An unpaired *t*‐test was used for unpaired comparisons. ANOVA analysis was used for between‐group comparisons, and the LSD test was used for further comparisons. The difference was statistically significant at *p <* 0.05.

## 3 Results

### 3.1 Immediate Histological Response in Classical Discharge Mode

**FIGURE 2:**
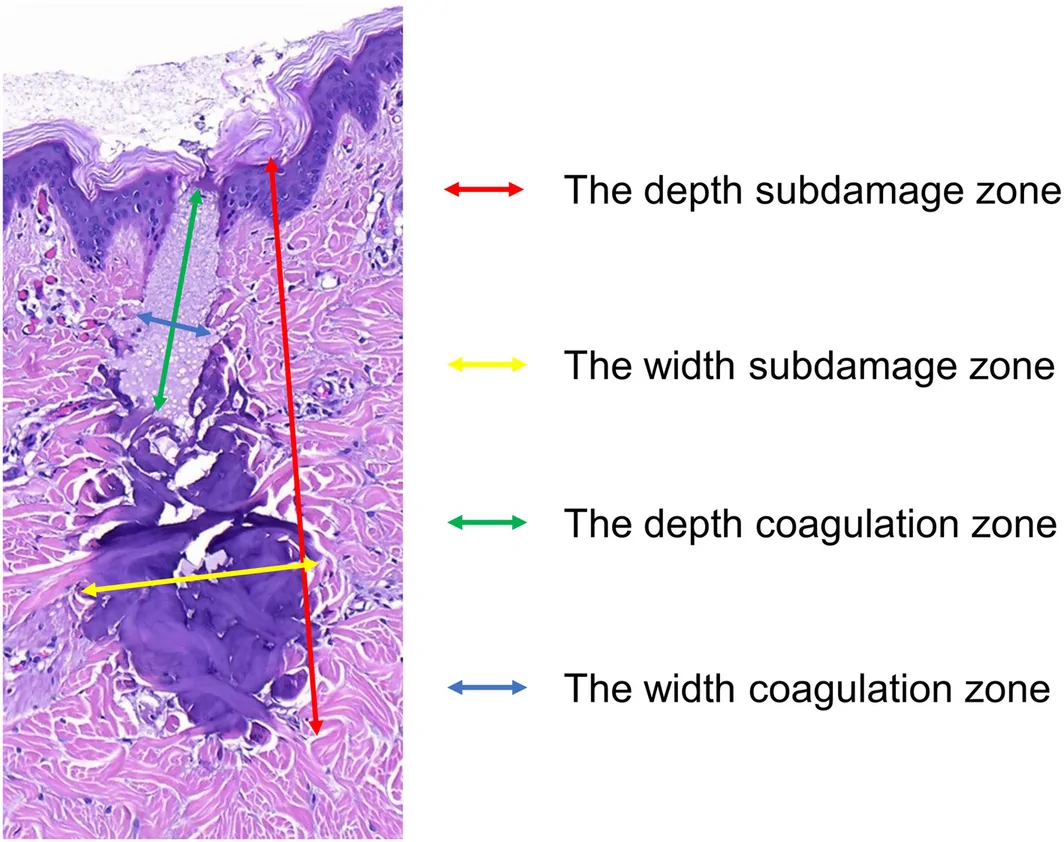
Schematic diagram of histological measurements.

**FIGURE 3:**
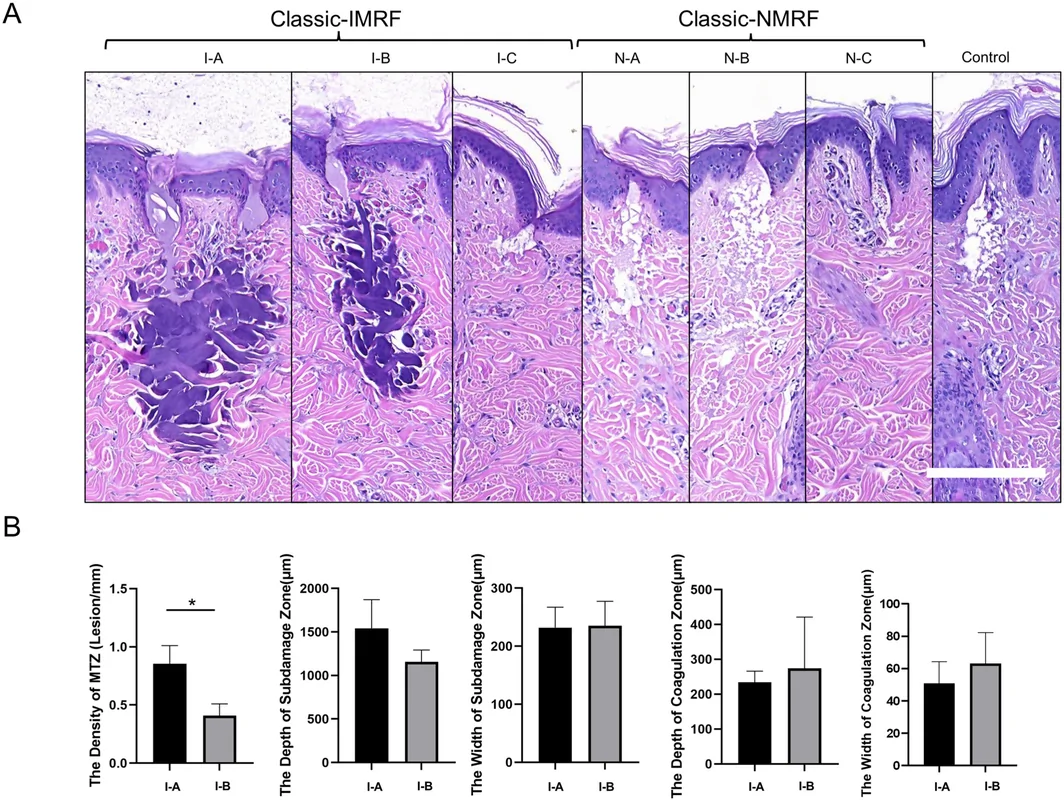
Histological observations of each group under the classic discharge mode. (A) HE staining immediately after radiofrequency microneedle treatment with different parameters under the classic discharge mode. Scale bar = 200 μm. (B) Quantitative histological statistics of insulated microneedles in the high‐power short‐pulse width group (I‐A) and in the medium‐power medium‐pulse width group (I‐B) under the classic discharge mode.

Under the classic discharge mode, all treated areas immediately exhibited slight erythema and pinpoint bleeding. There was no significant difference in the erythema reaction among the groups. The histological measurement scheme is shown in Figure 2. According to Figure 3A, no typical lesions were found in the classical discharge mode in the three groups of NMRF for all combinations of pulse width and power (N‐A, N‐B, N‐C), and no typical MTZs were found in the low power, long pulse width IMRF (I‐C, 5 W, 200 ms). Only the IMRF in the high power, short pulse width group (I‐A, 20 W, 50 ms) and in the medium power, medium pulse width group (I‐B, 10 W, 100 ms) can show MTZs. Therefore, only the two groups of classical discharge modes were selected for the statistics (Figure 3B). In these two IMRF groups, the lesion density was significantly higher in Group I‐A than in Group I‐B (*p* = 0.014) at the same total energy control and amounted to 0.86 ± 0.15/mm and 0.41 ± 0.10/mm, respectively. As for the depth and width of the sub‐damage zone and the depth and width of the coagulation zone, there was no significant difference between the two groups (I‐A and I‐B), as shown in Table 3.

**TABLE 3:** Quantitative histological partial statistics of insulated microneedles in the high‐power with short‐pulse width group (I‐A) and the medium‐power medium‐pulse width group (I‐B) under the classic discharge mode.

	I‐A	I‐B	*p*
The depth of sub‐damage zone (μm)	1542.96 ± 327.43	1157.33 ± 135.91	0.142
The width of sub‐damage zone (μm)	231.75 ± 35.28	235.33 ± 42.12	0.816
The depth of coagulation zone (μm)	233.75 ± 32.35	274.30 ± 146.61	0.185
The width of coagulation zone (μm)	50.81 ± 13.40	63.21 ± 19.05	0.714

### 3.2 Immediate Histological Response in Sequential Discharge Mode

**FIGURE 4:**
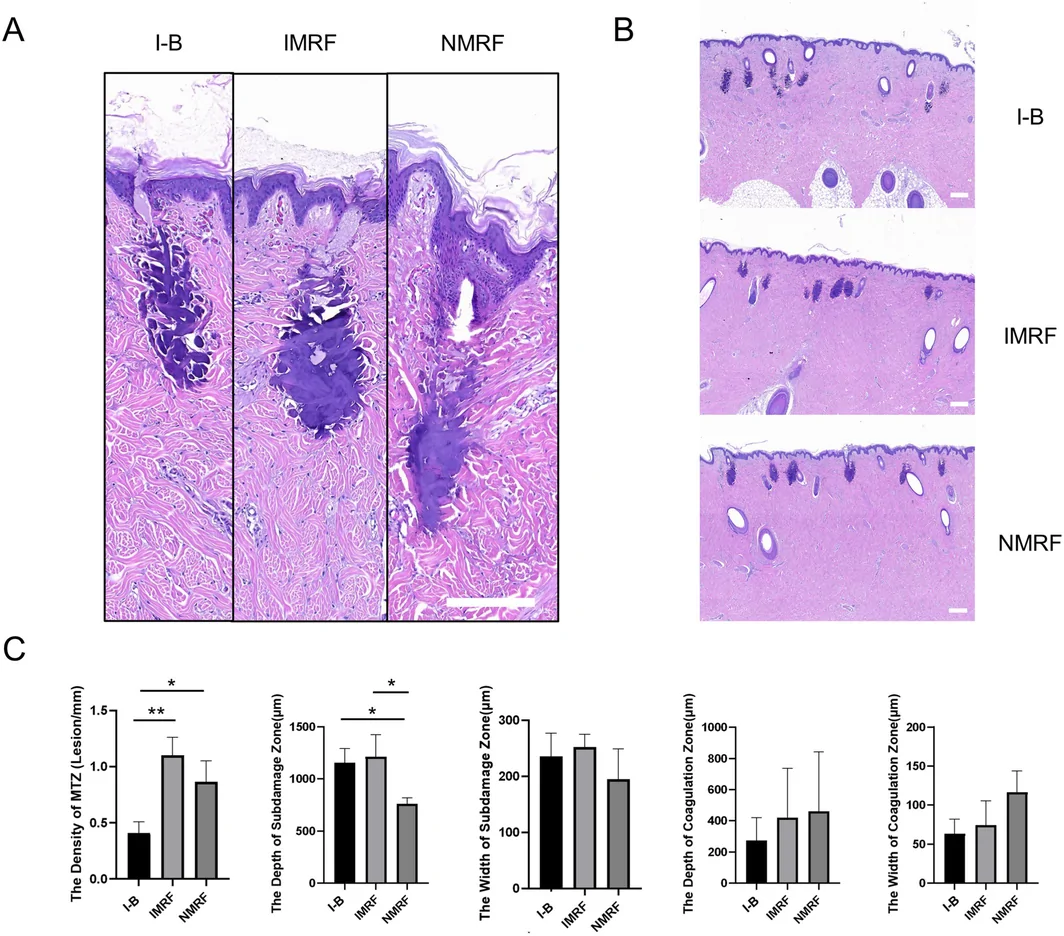
Histological observations of each group under the medium‐power medium‐pulse width under the two discharge modes. (A) HE staining immediately after MRF treatment with different parameters under the classic discharge mode. Scale bar = 200 μm. (B) Distribution of MTZ in the three groups. Scale bar = 300 μm. (C) Under the medium power and medium pulse width, the quantitative histological statistics of I‐B in the classical discharge mode, the IMRF and NMRF in sequential discharge mode.

The medium‐power, medium‐pulse‐width groups (I‐B and N‐B; 10 W, 100 ms) from the classical discharge mode were used as controls. Because no obvious MTZ was found in the N‐B group, it was excluded from statistical comparisons. We subsequently investigated both IMRF and NMRF in sequential discharge mode with the same parameters (10 W, 100 ms). Under the sequential discharge mode, each group showed a slight erythema and pinpoint bleeding. There was no significant difference in the erythema reaction among the groups. As illustrated in Figure 4A, MTZs were generated in both IMRF and NMRF under sequential discharge mode. By contrast, no MTZs could be detected in NMRF treated with classical discharge mode under identical parameters. Figure 4B,C further reveals that the MTZ density of both groups under sequential discharge mode was significantly higher than that of the classical discharge group (IMRF vs. I‐B *p* = 0.002, NMRF vs. I‐B *p* = 0.011). On the other hand, the depth of the sub‐damage zone of IMRF was significantly greater in sequential discharge mode than in classical discharge mode, and the depth of the sub‐damage zone was 1213.69 ± 210.66, 1157.33 ± 135.91, and 761.40 ± 57.17 μm, respectively (NMRF vs. I‐B *p* = 0.017, NMRF vs. IMRF *p* = 0.01). However, there was no significant difference in the width of the sub‐damage zone between the two types of IMRF (IMRF vs. I‐B *p* = 0.658). Thus, when the overall energy level remained unchanged, the actual power and pulse width of each pin varied with the discharge types. However, there is no significant difference in the width of the sub‐damage zone and the depth and width of the coagulation zone between the three groups of MRF (I‐B, NMRF, and IMRF).

### 3.3 Collagen Changes After MRF in Classical Discharge Mode

**FIGURE 5:**
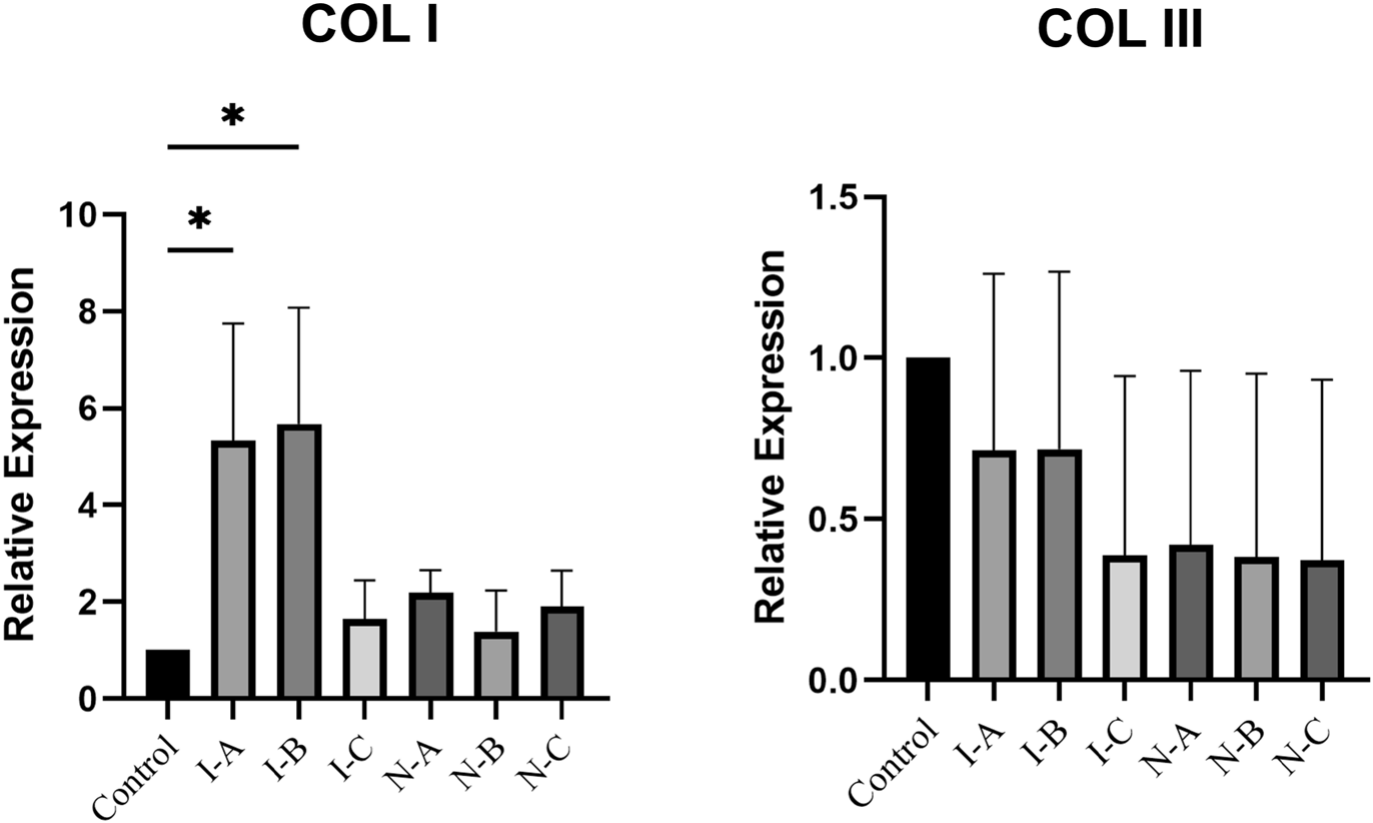
mRNA expression of COL I and COL III 3 months after treatment compared to the blank control group under the classic discharge mode.

At the 3‐month follow‐up, prior to tissue extraction, no PIH or other adverse events were macroscopically observed in any treatment areas. Subsequently, the expression of type I and type III collagen was analyzed with different parameters of IMRF and NMRF in the classical discharge mode. As shown in Figure 5, collagen type I was significantly upregulated in the high power, short pulse width group (I‐A) and in the medium power, medium pulse width group (I‐B) after treatment (I‐A *p* = 0.002, I‐B *p* = 0.001), while the other groups showed a slight upward trend but no significant difference compared to the empty control group. However, the expression of type III collagen was not significantly altered in all groups after treatment (*p* = 0.671).

## 4 Discussion

In this study, using an in vivo porcine skin model, we demonstrated that the critical determinant for generating effective MTZ of MRF was not merely the total energy input, but rather whether the “minimum power threshold per needle” is achieved. Under the controlled total energy conditions (1000 mJ), we identified significant limitations in conventional discharge mode. The NMRF, due to their larger active surface area and dispersed heat distribution, exhibit a substantially higher power threshold for MTZ formation compared to IMRF. It is understood that IMRF concentrated energy deposition exclusively at the needle tip, resulting in a smaller treatment volume, more focused heat accumulation, and consequently higher and faster tissue temperature rise, which facilitates MTZ formation. In contrast, the NMRF distribute energy along the entire needle shaft, leading to lower heat generation per unit length under equivalent energy per pulse. Therefore, NMRF require higher power settings to achieve MTZ formation. Clinically, this implies that power or energy parameters for NMRF should be set higher than those for IMRF in order to effectively generate MTZs and subsequently improve skin quality.

To break through the limitations of the above‐mentioned power threshold, we developed and introduced an innovative sequential discharge mode. Essentially, this mode is a combination of “ultra‐high power and ultra‐short pulse width.” Our histological quantitative analysis showed that, under the same moderate power setting, the sequential mode enables each microneedle to achieve an extremely high peak power instantaneously, thereby generating stable and dense tissue coagulation foci without increasing the total energy output. This indicates that the sequential discharge mode can significantly enhance the remodeling efficiency of the dermis without increasing the overall risk of thermal damage.

Previous studies have proved that the needle depth was related to the depth of the coagulation zone, considering that the microdefect was mainly caused by machinery [8]. Zheng et al. [7] have also suggested that changes in power or pulse width would affect the depth and width of the coagulation zone or sub‐damage zone. However, most of these conclusions were based on the premise that the total energy variable was not controlled. The rigor of this study lies in the control of the consistency of total energy. Under this condition, we found that once the minimum power threshold was crossed, an increase in the power of a single needle led to an increase in the density of the MTZ and a stabilization of its distribution, rather than an unlimited expansion of the MTZ's own “morphology” (depth/width). This finding provides an important theoretical correction for the precise parameter adjustment in clinical practice.

Finally, the histological MTZ must be transformed into long‐term collagen neogenesis to have clinical significance. We found that the high‐power and medium‐power groups, which were capable of generating typical MTZ, all showed a significant upregulation of type I collagen at 3 months post‐treatment. Given the established correlation between MTZ formation and long‐term type I collagen neogenesis observed in the classical discharge mode, it is reasonable to hypothesize that the sequential discharge mode—which demonstrated a significantly higher MTZ density in immediate histology—holds substantial clinical antiaging potential. However, since long‐term molecular data for the sequential mode were not directly evaluated in this study, whether it translates to a significantly more potent dermal remodeling effect compared to the classical mode remains to be confirmed by future comparative investigations.

## 5 Conclusion

Based on the live pig model, we concluded that the generation of MTZ by MRF is highly dependent on the minimum power threshold of a single needle rather than the total energy alone. Moreover, the effective power threshold of NMRF is significantly higher than that of IMRF. Compared with the classic discharge mode, the innovative sequential discharge mode, through an output mechanism of “ultra‐high power and ultra‐short pulse width,” effectively formed denser and more stable MTZs in the dermis without increasing the total energy. Given that MTZ generation essentially drives collagen neogenesis, this sequential mode exhibits promising clinical potential with low thermal risk, representing a pivotal direction for future MRF optimization.

## Author Contributions

J.F., X.W., L.Z., S.M., and L.H. performed the research. J.F. and L.H. designed the research study. J.F., X.W., L.Z., and S.M. analyzed the data. J.F. wrote the paper.

## Conflicts of Interest

The authors declare no conflicts of interest.

## Data Availability

The data that support the findings of this study are available from the corresponding author upon reasonable request.
